# CD33^+^/p-STAT1^+^ double-positive cell as a prognostic factor for stage IIIa gastric cancer

**DOI:** 10.1007/s12032-012-0442-2

**Published:** 2013-01-11

**Authors:** Jun Dong, Jiao Li, Shi-Ming Liu, Xing-Yu Feng, Shi Chen, Ying-Bo Chen, Xiao-Shi Zhang

**Affiliations:** 1 State Key Laboratory of Oncology in South China; Biotherapy Center, Cancer Center, Sun Yat-sen University Cancer Center, No. 651, Dongfeng Road East, Guangzhou, 510060 Guangdong Province China; 2 Department of Cardiology, Guangzhou Institute of Cardiovascular Disease, Second Affiliated Hospital of Guangzhou Medical University, Guangzhou, 510260 Guangdong Province China; 3 State Key Laboratory of Oncology in South China, Department of Gastric & Pancreatic Surgery, Sun Yat-sen University Cancer Center, Guangzhou, 510060 Guangdong Province China

**Keywords:** Gastric cancer, CD33, STAT1, T cell, B cell, Regulation

## Abstract

Tumor-infiltrating immune cells are associated with tumor prognosis, although the type of immune cells responsible for local immune escape is still unknown. This study examined the relationship between gastric cancer survival and the density of immune cells, including CD8^+^ T cells, CD20^+^ B cells, and CD33^+^/p-STAT1^+^ cells, which represent myeloid-derived suppressor cells, to evaluate the role of immune cells in the progression of gastric cancer. One hundred pathologically confirmed specimens were obtained from stage IIIa gastric cancers between 2003 and 2006 at Sun Yat-sen University Cancer Center, China. The density of tumor-infiltrating immune cells in tumor tissue was examined using immunohistochemical analysis. Clinicopathologic parameters and the survival rate were analyzed in relation to the density of immune cells. A high density of CD8^+^ T cells and CD20^+^ B cells was associated with a good clinical outcome, but a high density of CD33^+^/p-STAT1^+^ cells was associated with a poor clinical outcome. Most importantly, the density of CD33^+^/p-STAT1^+^ cells was an independent prognostic factor and inversely related to the infiltration of CD8^+^ T cells. Although the infiltration of CD8^+^ T cells and CD20^+^ B cells is involved in the progression of gastric cancer, these data suggest that CD33^+^/p-STAT1^+^ cells play a central role in the regulation of the local immune response, suggesting that CD33^+^/p-STAT1^+^ cells might be therapeutic targets in gastric cancer.

## Introduction

Gastric cancer is one of the most aggressive diseases worldwide, particularly in Asian countries, such as China [1]. In addition, gastric cancer is the second leading cause of cancer-related death each year due to the resistance and intolerance of this cancer to cytotoxic therapy [2–4]. New therapeutic strategies are needed to treat gastric cancer. Recently, immunotherapy has received increased attention because antibodies targeting immune checkpoint molecules and therapeutic vaccines have been identified [5–8]; therefore, identifying patients who are suitable for immune therapy is critical.

The density of tumor-infiltrating immune cells is associated with the prognosis of multiple types of tumors [9–13]. However, tumor-infiltrating immune cells consist of both tumor-rejecting cells and tumor-promoting cells. Therefore, a detailed analysis of tumor-infiltrating immune cells would lead to more effective prognostic indicators, and their potential use as biomarkers for immunotherapy could be determined [14, 15].

Immune cells infiltrates are different among tumor types and from patient to patient. Tumor-infiltrating lymphocytes (TILs) are considered to be a manifestation of the host immune reaction to cancer cells [16]. Large numbers of T and B lymphocytes are associated with a good clinical outcome in many different tumor types [13, 17–26].

In addition to tumor-rejecting immune cells, immune suppressor cells play a pivotal role in tumor progression. Recently, several studies have demonstrated the importance of myeloid-derived suppressor cells (MDSCs) in tumor-associated immune suppression [27, 28]. MDSCs are recognized as a heterogeneous population of myeloid cells that consist of immature myeloid cells (IMCs) and myeloid cells at early stages of differentiation [27, 29, 30]. The role of the MDSCs that infiltrate into solid tumor tissue is still unclear. An obstacle to assaying the role of MDSCs in human tissue is the lack of defined markers for MDSCs. Among the markers reported, CD33 and CD11b are the basic markers of myeloid-derived suppressor cells. Although they are feasible to assay in blood cells, staining cells in tissue for multiple markers is very difficult, especially for markers with the same subcellular localization. To develop a set of markers that are suitable for the detection of infiltrative MDSCs, we reviewed the mechanisms of activation and expansion of MDSCs. The expansion and activation of MDSCs are influenced by several different factors, including VEGF, GM-CSG, SCF, TGF-β, and MMP9, among others [31–39]. These factors trigger the activation of several different signaling pathways in MDSCs that promote JAK-STAT signaling, including STAT1, STAT3, and STAT6 [28, 40]. To investigate this role, CD33 was used as a marker of immature myeloid cells, and p-STAT1 was used as a marker of IMCs activated by immune regulatory cytokines [40, 41]. Therefore, CD33^+^/p-STAT1^+^ cells were proposed to be a subset of MDSCs in gastric cancer tissues. This study examined the relationship between the survival rate of gastric cancer and the density of immune cells, including TILs and CD33^+^/p-STAT1^+^ cells, which represented MDSCs, to evaluate the role of the immune response in gastric cancer progression.

## Materials and methods

### Tissue specimens

Formalin-fixed, paraffin-embedded tissues from 100 gastric cancer patients were used. Gastric cancer biopsy specimens were collected from stage IIIa (2009 UICC staging system) gastric cancer patients between 2003 and 2006 at the Sun Yat-sen University Cancer Center. All of the patients underwent radical resection, and none of the patients had chemotherapy or radiotherapy before sample collection. Patients received 5-FU-based adjuvant chemotherapy post-operatively for 6 months. If recurrence or metastasis occurred, 5-FU-based chemotherapy was given according to the NCCN guidelines. This study was conducted in accordance with the Declaration of Helsinki, and all patients signed a consent form approved by the Research Ethics Committee of the Sun Yat-sen University Cancer Center.

### Immunohistochemistry and scoring systems

Paraffin-embedded tissues were sectioned continuously at a thickness of 4 μm and heated for 1 h at 65 °C. Briefly, the sections were deparaffinized using xylenes and rehydrated with a graded alcohol series and distilled water. The sections were immersed in an EDTA antigen retrieval buffer (pH 8.0), subjected to high pressure for 3 min for antigen retrieval and allowed to cool to room temperature. After blocking with sheep serum, the sections were incubated overnight at 4 °C with mouse monoclonal antibodies against human CD8, CD20, and GrB (Zymed, San Diego, CA, USA), all of which were diluted 1:400. Following incubation with secondary antibodies, the sections were developed using diaminobenzidine tetrahydrochloride (DAB) and counterstained with hematoxylin.

The co-expression of CD33 and p-STAT1 was detected with sequential immunohistochemical staining using the EnVison™ G/2 Doublestain System (Dako Cytomation, Glostrup, Denmark) according to the manufacturer’s instructions. Endogenous peroxidases and alkaline phosphatase enzymes were blocked with the dual endogenous enzyme-blocking reagent provided in the kit. The sections were treated with normal goat serum for 20 min to reduce nonspecific binding and incubated overnight at 4 °C with rabbit polyclonal anti-CD33 antibody (1:100; Protein Tech Group, Chicago, IL, USA) and rabbit monoclonal anti-p-STAT1 (1:400; Cell Signaling, Boston, MA, USA). For the color reaction, diaminobenzidine (brown) and permanent red (red) were must used. As a negative control, the antibodies were replaced with phosphate-buffered saline (PBS).

The density of immune cells within the tumor specimens was scored as we previously reported [14]. Briefly, the number of cells and the cell size were counted in at least 10 different fields of each section. The size of each high-powered field (400×) was approximately 300 μm × 300 μm, and the cells were counted in the intratumoral compartment. The areas of highest density were chosen, and necrotic areas were avoided. Two observers counted the cells at the same time in the same field using a multiple-lens microscope. The median value was used to distinguish the different groups of immunohistochemical variables in the results.

### Statistical analysis

All statistical analyses were performed with the SPSS 16.0 statistical software package. The median value was used to differentiate high- and low-density groups for each immunohistochemical variable. The correlation between the density of immune cells and patient characteristics and the correlation between the density of TILs and CD33^+^/p-STAT1^+^ cells were analyzed using correlation test. Kaplan–Meier curves were used to estimate the distribution of variables in relation to survival, which were compared using the log-rank test. Univariate and multivariate analyses were based on the Cox proportional hazards regression model. Overall survival (OS) was defined as death from any cause, and disease-free survival (DFS) was defined as the time prior to relapse of the primary tumor*. p* < 0.05 was considered to be statistically significant.

## Results

### Patient characteristics

Among the 100 patients, there were 71 men and 29 women, and the median age was 59.5 years, with a range of 29–82 years. Fifty-five patients (55 %) were Borrmann classification II, 38 were Borrmann classification III, and 7 cases were Borrmann classification I or IV. All of the patients presented lymph node metastasis before treatment, with 36 patients at the N1 stage, 34 patients at the N2 stage, and 30 patients at the N3 stage. During follow-up, 54 died, and 44 presented with disease progression.

### CD8, CD20, GrB, and CD33^+^/p-STAT1^+^ cells in gastric cancer tissues

**Fig. 1:**
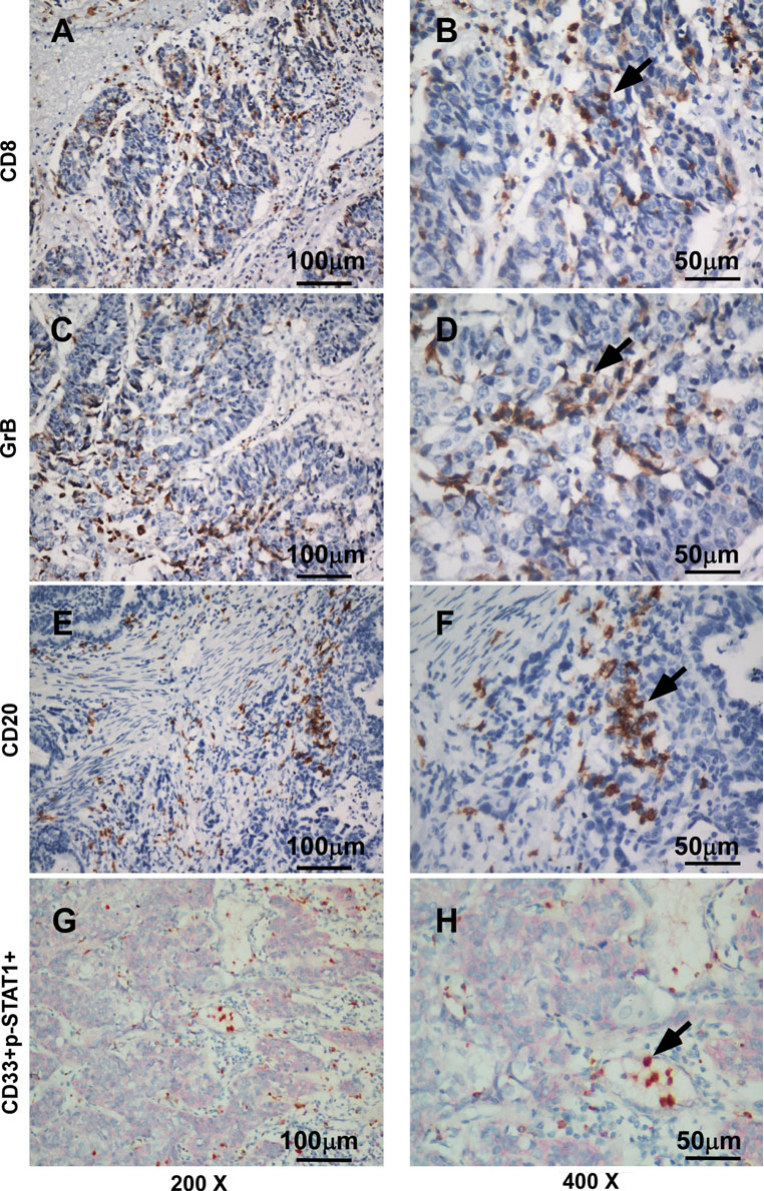
Immunohistochemical staining for CD8, CD20, GrB, and double immunohistochemical staining for CD33 and p-STAT1 (200× and 400×). **a**, **b** CD8^+^ T lymphocytes in gastric cancer tissue. **c**, **d** GrB^+^ active cytotoxic T lymphocytes in gastric cancer tissue. **e**, **f** CD20^+^ B lymphocytes in gastric cancer tissue. **g**, **h** Gastric cancer tissue stained for CD33^+^ (*red*) and p-STAT1^+^ (*brown*)

CD8^+^, CD20^+^, and GrB^+^ lymphoid cells displayed strong membrane staining (Fig. 1a–f). CD8^+^ T cells and CD20^+^ B cells were primarily observed in cancer nests and along the invasive margin. The infiltrating lymphoid cells in the intratumoral compartment were counted.

To characterize MDSC infiltration in gastric cancer tissue, we defined MDSCs as CD33^+^/p-STAT1^+^ cells. These CD33^+^/p-STAT1^+^ double-positive cells have been found mostly in the stroma among gastric cancer (Fig. 1g, h), whereas they had not yet been observed in the normal mucosa adjacent to gastric cancer and normal mucosa far from the gastric cancer (data not shown). The CD33 immunostaining demonstrated cytomembrane staining, and p-STAT1 demonstrated nucleus staining. Figure 1g, h shows the CD33/p-STAT1 double-positive cells in a subset of cells around the tumor nests.

### Relationship between the density of CD33^+^/p-STAT1^+^ cells and the density of CD8^+^ and CD20^+^ lymphocytes

The correlation test was used to compare the density of CD33^+^/p-STAT1^+^ cells with the density of CD8^+^ and CD20^+^ lymphocytes (Table 1). A higher density of CD33^+^/p-STAT1^+^ cells within tumor nests was associated with a lower density of CD8^+^ T cells (Spearman’s rho = −0.538, *p* < 0.001). However, CD33^+^/p-STAT1^+^ cells were not significantly associated with the density of CD20^+^ B lymphocytes (Spearman’s rho = −0.036, *p* = 0.721).

**Table 1:** Relationship between CD33^+^/p-STAT1^+^ cells and the density of TILs

	CD33^+^/p-STAT1^+^ cells	
	Spearman’s rho	*p* value
Density of CD8^+^ T cells	−0.538	<0.001
Density of CD20^+^ B cells	−0.036	0.721

### Relationship between the density of CD8, CD20, and CD33^+^/p-STAT1^+^ cells and clinicopathologic characteristics

The immune cells were divided into two groups based on the median value (high density and low density). The cutoff value for the density of CD8, CD20, and CD33^+^/p-STAT1^+^ groups was 28, 34, and 11 cells, respectively, per high-powered field in the center of the tumor. The density of immune cells was analyzed to identify any association with the clinicopathologic features of gastric cancer. As shown in Table 2, the density of CD8^+^ T cells and CD33^+^/p-STAT1^+^ cells was significantly correlated with patient vital status (*p* = 0.023, *p* < 0.001) and relapse occurrence (*p* = 0.005, *p* = 0.001); in addition, the high-density group of CD8^+^ T cells had a smaller tumor size (*p* = 0.005). However, the density of the immune cells was not significantly associated with gender, age, or Borrmann classification.

**Table 2:** Correlation between clinicopathologic features and immune cell density

Characteristics	CD8^+^ T lymphocytes			CD20^+^ B lymphocytes			CD33^+^/p-STAT1^+^ cells		
	High density	Low density	*p*	High density	Low density	*p*	High density	Low density	*p*
	(*n* = 38)	(*n* = 62)		(*n* = 43)	(*n* = 57)		(*n* = 43)	(*n* = 57)	
Gender									
Male	31	40	0.068	32	39	0.513	28	43	0.260
Female	7	22		11	18		15	14	
Age (year)									
<60	11	17	0.869	10	18	0.359	14	14	0.378
≥60	27	45		33	39		29	43	
Borrmann classification									
I	2	2	0.554	2	2	0.884	1	3	0.950
II	23	32		22	33		24	31	
III	13	25		18	20		17	21	
IV	0	3		1	2		1	2	
Tumor size (cm^3^)									
<20	25	23	0.005	20	28	0.796	22	26	0.582
≥20	13	39		23	29		21	31	
Vital status									
Alive	23	23	0.023	19	27	0.752	9	37	<0.001
Dead	15	39		24	30		34	20	
Relapse									
Yes	10	34	0.005	18	26	0.708	27	17	0.001
No	28	28		25	31		16	40	

### Association of the density of immune cells with patient survival

**Fig. 2:**
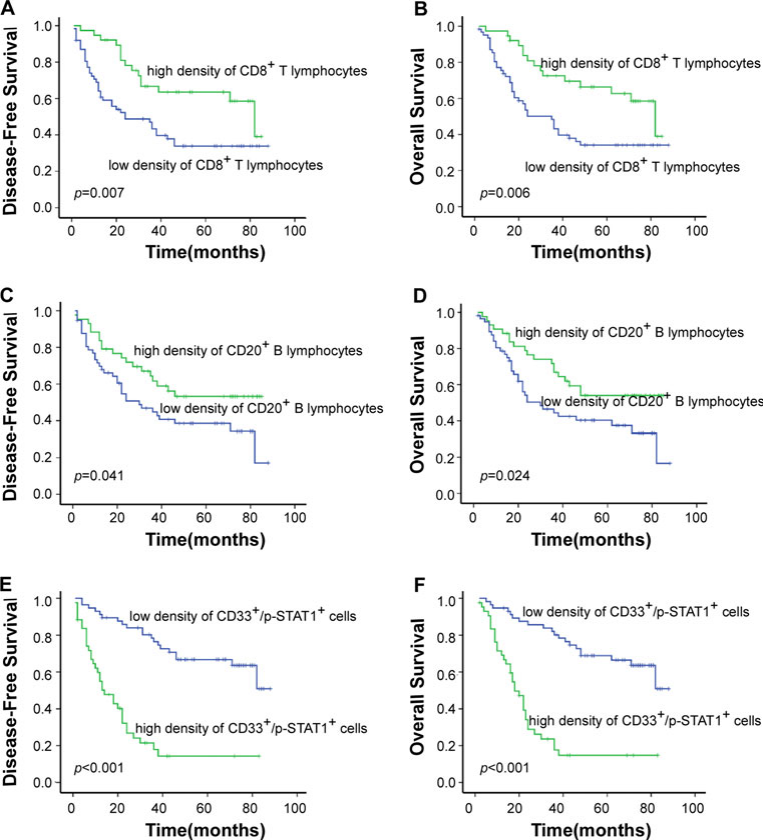
Kaplan–Meier analysis of disease-free survival and overall survival for each immune cell group. A high density of CD8^+^ T lymphocytes and CD20^+^ B lymphocytes was associated with a longer overall survival and longer disease-free survival than a low density, but the inverse result was observed for CD33^+^/p-STAT1^+^ cell density

The median follow-up time was 36.5 months, with a range of 2–88 months. The median DFS was 32.5 months, with a range of 2–88 months. At the completion of the study, 46 patients with stage IIIa gastric cancer were alive, whereas 54 patients had died. Fifty-two deaths were cancer related, and two deaths were unrelated to cancer. The cumulative 5-year survival rate was 32.0 % for all patients with stage IIIa gastric cancer. We evaluated whether the density of immune cells was associated with patient prognosis. The disease-free survival time differed significantly between the immune cell groups (CD8^+^ T cells: *p* = 0.007; CD20^+^ B cells: *p* = 0.041; CD33^+^/p-STAT1^+^ cells: *p* < 0.001; Fig. 2). Patients with a high density of lymphocytes had a longer DFS time than those with low density. We next analyzed the association between the density of immune cells and OS time (CD8^+^ T cells: *p* = 0.006; CD20^+^ B cells: *p* = 0.024; CD33^+^/p-STAT1^+^ cells: *p* < 0.001; Fig. 2). The cumulative 5-year survival rate was 50.9 % in the low-density CD33^+^/p-STAT1^+^ group but only 7.0 % in the high-density CD33^+^/p-STAT1^+^ group.

Univariate analysis demonstrated that age, and the density of CD8, CD20, and CD33^+^/p-STAT1^+^ cells were significant prognostic factors for OS and DFS (Table 3). However, clinical prognosis was not associated with gender, age, Borrmann classification, size of the tumor, or N stage.

**Table 3:** Univariate analysis of factors associated with OS and DFS

Variables	OS (*n* = 100)		DFS (*n* = 100)	
	HR (95 % CI)	*p* value	HR (95 % CI)	*p* value
Gender	1.636 (0.934–2.864)	0.085	1.587 (0.906–2.778)	0.106
Age	1.903 (1.094–3.311)	0.023	1.946 (1.118–3.390)	0.019
Borrmann classification	1.124 (0.750–1.685)	0.570	1.129 (0.751–1.696)	0.561
Size of tumor	1.255 (0.733–2.148)	0.408	1.278 (0.746–2.187)	0.372
N stage	0.828 (0.586–1.169)	0.283	0.835 (0.594–1.175)	0.301
CD8^+^ T cells	0.446 (0.245–0.811)	0.008	0.452 (0.249–0.821)	0.009
CD20^+^ B cells	0.533 (0.304–0.934)	0.028	0.566 (0.323–0.991)	0.046
CD33^+^/p-STAT1^+^ cells	5.318 (2.965–9.538)	<0.001	5.330 (2.971–9.561)	<0.001

### Multivariate Cox proportional hazard analysis

A multivariate Cox proportional hazard analysis was performed for age, and the density of CD8, CD20, and CD33^+^/p-STAT1^+^ cells. Of the 100 stage IIIa gastric cancer patients, the Cox regression model revealed that only patients with a higher density of CD33^+^/p-STAT1^+^ cells had a significantly reduced OS (hazard ratio [HR]: 4.674; 95 % CI: 2.525–8.625) and DFS (HR: 4.670; 95 % CI: 2.537–8.596) compared to the low CD33^+^/p-STAT1^+^ group. However, CD8 and CD20 were not associated with OS or DFS (Table 4).

**Table 4:** Multivariate analysis of factors associated with OS and DFS

Variables	OS (*n* = 100)		DFS (*n* = 100)	
	HR (95 % CI)	*p* value	HR (95 % CI)	*p* value
Age	1.515 (0.857, 2.677)	0.153	1.603 (0.903, 2.846)	0.107
CD8^+^ T cells	0.595 (0.321, 1.101)	0.098	0.571 (0.309, 1.054)	0.073
CD20^+^ B cells	0.555 (0.312, 0.986)	0.045	0.632 (0.356, 1.121)	0.116
CD33^+^/p-STAT1^+^ cells	4.674 (2.525, 8.652)	<0.001	4.670 (2.537, 8.596)	<0.001

## Discussion

To enhance our understanding of the contribution of the local immune response to the progression of gastric cancer, this study characterized CD8^+^ T lymphocytes, CD20^+^ B lymphocytes, and CD33^+^/p-STAT1^+^ cells in the intratumoral region. The results showed that the density of CD8^+^ T cells, CD20^+^ B cells, and CD33^+^/p-STAT1^+^ cells in tumors was a useful criterion for the prediction of survival in advanced gastric cancer patients. Most importantly, Cox multivariate analysis demonstrated that the density of CD33^+^/p-STAT1^+^ cells within the cancer tissue was an independent prognostic factor. These data indicated that CD33^+^/p-STAT1^+^ cells play an important role in the progression of gastric cancer.

Several recent studies have analyzed the important role of tumor-infiltrating cytotoxic CD8^+^ T and B lymphocytes in the antitumor immune response [42–49]. The effects of CD8^+^ T cells in cancer cell nests might be related to the effector function of killer T cells, and the role of B lymphocytes as part of the adaptive humoral immune response has been associated with improved survival in several types of cancer [25, 26, 50].

Although some studies have indicated that immune surveillance and immune escape exist in gastric cancer [51–53], the type of immune cells responsible for local immune escape is still unknown. Experimental data indicated that MDSCs suppress the immune response, in contrast to CD8^+^ T cells and CD20^+^ B cells. The main obstacle is the markers for MDSCs in the human tissue. As mentioned above, besides the basic markers of human MDSCs, the signaling pathways associate with the MDSCs expansion and activation are also very important. STAT1 is one of the pathways in MDSCs activation; Gabrilovich demonstrated that STAT1 activation in tumor-associated macrophages is responsible for the upregulation of inducible nitric oxide synthase (iNOS) and arginase 1 activity in these cells, which results in T-cell suppression [41]. STAT1-deficient MDSCs are unable to inhibit T-cell activation due to their inability to upregulate iNOS and arginase 1 activity [54, 55]. Therefore, CD33 staining on the cell membrane was used as a marker of myeloid cells in this study, and p-STAT1 nuclear staining was used to represent a transcription factor activated by pro-inflammatory cytokines. For this reason, CD33^+^/p-STAT1^+^ cells are defined as a specific type of MDSC in gastric cancer tissues, although further functional analysis is required. Using this set of MDSC markers, it was possible to examine the infiltration of MDSCs into cancer tissues.

To confirm the role of CD33^+^/p-STAT1^+^ cells, which represented MDSCs in gastric cancer tissue, this study detected the density of CD8^+^ T cells, CD20^+^ B cells, and CD33^+^/p-STAT1^+^ immune cells infiltrating tumor tissue. A higher density of CD33^+^/p-STAT1^+^ cells was inversely related to infiltrating CD8^+^ T lymphocytes but not B cells, supporting that CD33^+^/p-STAT1^+^ cell might be a subset of MDSCs in gastric tumor tissue. Although Kaplan–Meier analysis and log-rank tests revealed that the all of the immune cells were significantly associated with OS and DFS duration, Cox multivariate analysis demonstrated that only the density of CD33^+^/p-STAT1^+^ cells within the cancer tissue was a prognostic factor for OS and DFS, independently, indicating that CD33^+^/p-STAT1^+^ cells play a key role in the regulation of the local immune response.

Overall, this study demonstrated that the density of CD33^+^/p-STAT1^+^ cells is an independent prognostic factor for patients with stage IIIa gastric cancer, indicating that CD33^+^/p-STAT1^+^ cells play a central role in the local immune response in gastric cancer. Therefore, CD33^+^/p-STAT1^+^ cells might be a useful therapeutic target.
